# A data-driven performance index for center forwards in the English Premier League

**DOI:** 10.1038/s41598-026-55681-9

**Published:** 2026-06-08

**Authors:** Pablo Prieto-González, Fatma Hilal Yagin, Víctor Martín, Rui Marcelino, Abdullah F. Alghannam, Burak Yagin, Alejandro Sal-de-Rellán

**Affiliations:** 1 Sport Sciences and Diagnostics Research Group, College of Humanities and Sciences, Prince Sultan University, Riyadh, Saudi Arabia; 2 Department of Biostatistics, Faculty of Medicine, Malatya Turgut Ozal University, Malatya, 44280 Turkey; 3 Faculty of Health Sciences, Universidad Isabel I de Castilla, Burgos, Spain; 4 Research Center in Sports Sciences, Health Sciences and Human Development, CIDESD, Elite Research Community, Maia, Portugal; 5 University of Maia, Maia, Portugal; 6 FPF Academy, Portuguese Football Federation, Oeiras, Portugal; 7 Lifestyle & Health Research Center, Princess Nourah bint Abdulrahman University, Riyadh, Saudi Arabia; 8 Department of Biostatistics and Medical Informatics, Faculty of Medicine, Inonu University, Malatya, 44210 Turkey; 9 Department of Education and Educational Innovation, Faculty of Law, Education and Humanities, Universidad Europea de Madrid, Madrid, Spain

**Keywords:** Soccer performance index, Center forwards, Performance analysis, Regression analysis, Market value, Talent identification, Health care, Mathematics and computing

## Abstract

Despite the growing availability of performance data in professional soccer, existing player rating systems lack positional specificity and fail to capture the multidimensional demands of center forwards. Building on composite index and regularized regression approaches in sports analytics, this study develops a Soccer Performance Index (SPI) tailored to center forwards in the English Premier League (EPL) across the 2021–2024 seasons. Data from 194 player-season observations were analysed using 109 Wyscout performance metrics as predictors. Three SPI versions were constructed using Lasso and Ridge regression: one based on market value, one on the InStat Index, and a hybrid combining both. The hybrid model, weighting market value at 70% and the InStat Index at 30%, achieved the strongest explanatory fit (R^2^ = 0.676, RMSE = 0.669), accounting for approximately 68% of variance in player valuation — a result consistent with the complexity inherent in behavioural and performance modelling contexts. xG per 90, shots, and key passes per 90 emerged as the strongest predictors. Methodological considerations include the use of a minimum participation threshold (≥ 20 matches), which may introduce survivorship bias; the treatment of player-season observations as independent units, which does not account for repeated-measures dependence; and the reliance on internal validation only. The SPI demonstrates ecological relevance by integrating both financial and on-field performance indicators, offering a structured framework for talent identification and recruitment support in applied professional contexts.

**Supplementary Information:**

The online version contains supplementary material available at 10.1038/s41598-026-55681-9.

## Introduction

Evaluating the performance of center forwards in professional soccer extends well beyond goal-scoring statistics. While goals remain the most visible output, modern tactical demands require these players to contribute actively to build-up play, create chances for teammates, and adapt to different tactical systems^1–3^. Center forwards occupy a uniquely demanding role: they operate under constant defensive pressure, must combine goal-seeking movements with key link-up actions, and are required to create scoring conditions through individual skill and positional intelligence^4^. Empirical evidence confirms that forwards present a distinct technical and tactical profile from all other positional groups, with the largest performance differences observed precisely between forwards and central defenders^5^; their degree of prominence further fluctuates depending on the tactical scheme adopted^6,7^ underscoring the need to analyse a broad range of factors when evaluating their effectiveness. Despite this multidimensional reality, most existing player rating systems assign a single composite score regardless of playing position, relying heavily on collective metrics that do not adequately capture position-specific contributions^8–10^.

Advances in sports analytics have made increasingly objective, data-based evaluations of individual performance possible^11^, enabling researchers to move beyond traditional metrics such as goal counts and incorporate more holistic indicators of a player’s contribution^7^. The English Premier League (EPL), renowned for its competitiveness and high-intensity style of play^12^, provides an ideal setting for this type of analysis given the particular physical, technical, and tactical demands it places on center forwards^13,14^. Early work by McHale and Scarf^9^ on the EA Sports Player Performance Index and the adaptation of Elo-based systems to team sports^10^ represented important steps, but these approaches share a fundamental limitation: they are not designed to reflect the specific role and responsibilities of center forwards. More recent comparisons of commercial rating systems such as WhoScored, FotMob, and Sofascore^8^ have confirmed that, despite progress in single-value rankings, positional differentiation and sensitivity to individual impact remain significant shortcomings. However, none of these systems reports psychometric validity, applies position-specific variable selection, or undergoes external validation — methodological requirements that the present study aims to address.

Soccer analytics has grown substantially as a research field over the past decade, driven by the exponential increase in available tracking and event data. Cefis^15^ conducted a comprehensive bibliometric analysis of the soccer analytics literature between 2010 and 2020, highlighting that while performance evaluation and machine learning applications have emerged as dominant themes, position-specific modeling remains underdeveloped. Within this broader landscape, the development of composite performance indicators has gained methodological traction. Cefis and Carpita^16^ applied a third-order PLS-SEM confirmatory approach to construct role-specific composite indicators using EA Sports data across the top five European leagues, demonstrating that performance sub-dimensions carry different significance weights depending on playing position and providing a rigorous methodological benchmark for position-specific index construction. However, their approach is inherently confirmatory: PLS-SEM requires a pre-specified theoretical structure and does not perform data-driven variable selection, limiting its applicability in high-dimensional exploratory settings such as the present study. Furthermore, advances in predictive modeling tools such as expected goals (xG) — examined in depth by Cefis and Carpita^17^ in terms of accuracy and explainability across statistical and machine learning classifiers — underscore the need for transparent, interpretable models that communicate clearly which performance dimensions drive the index. Despite these advances, no validated, position-specific performance index for EPL center forwards has been developed combining regression-based modeling with both advanced performance metrics and financial valuation data. Collectively, these contributions confirm the methodological viability of position-specific composite indices but leave an important gap: to the best of our knowledge, no study has combined regularized regression modeling with both advanced performance metrics and financial valuation data to construct a position-specific index for EPL center forwards.

Research on the relationship between on-field metrics and market value has consistently shown that goal-scoring and creative indicators are among the strongest predictors of player valuation^18,19^, particularly in the EPL, where striker market values are the highest among the top five European leagues. However, these studies have not translated their findings into a structured, position-specific predictive index. The present study addresses this gap by developing a Soccer Performance Index (SPI) specifically for center forwards in the EPL across the 2021–2024 seasons. The SPI is grounded in an empirically supported performance framework that conceptualizes individual contribution along four theoretically distinct dimensions. Finishing ability (goals, shots, xG per 90) reflects the primary output demand of the position, consistent with evidence that shooting volume and accuracy are among the strongest discriminators of success in professional soccer^20^. Playmaking and creative contribution (key passes, assists, xA per 90) captures the link-up role increasingly demanded of modern center forwards^21^. Duel and physical performance (aerial duels, offensive duels won) reflects the contest-based demands inherent to the position. Attendance consistency (matches played, minutes played) accounts for player availability as a performance moderator, consistent with position-specific style characterization approaches^22^. The SPI is constructed using Lasso and Ridge regression applied to 109 Wyscout performance metrics, enabling data-driven variable selection in a high-dimensional setting. Lasso was selected for its capacity to perform automatic variable selection by shrinking redundant coefficients exactly to zero, while Ridge was selected for its superior coefficient stability under high multicollinearity — an expected feature of correlated performance metrics. Elastic Net, which combines both penalties through an additional mixing hyperparameter, was considered but not adopted: in sparse, high-dimensional settings where the analytical goal is to identify individual predictive metrics rather than grouped variable clusters, the L1 penalty of Lasso already provides sufficient shrinkage and selection; introducing the additional α hyperparameter would require a further cross-validation layer that is statistically unjustified at this sample size and would reduce interpretability without a corresponding gain in predictive accuracy. Three SPI variants are developed: one based on market value, one on the InStat Index, and a hybrid model combining both. Market value serves in this study as an external criterion variable for index construction, not as a measure of on-field performance per se; it is incorporated as a widely accepted proxy for overall player valuation^18,19^, with the understanding that it also reflects contextual factors beyond match performance, such as age, contract status, and economic conditions.

Two specific hypotheses are tested: *H*_*1*_: regularized variable selection will reveal that technical finishing and playmaking indicators outweigh physical and spatial efficiency metrics, establishing that positional performance constructs are independent of market-driven popularity; *H*_*2*_: an integrated framework combining pure performance tracking (InStat Index) and economic valuation (market value) will explain a significantly greater proportion of variance than isolated single-indicator models, operationalizing the latent synergy between on-pitch utility and institutional market signals. The findings aim to contribute a methodological framework for forward evaluation and talent identification in the EPL that may inform data-driven recruitment decisions, subject to validation in independent samples^23^.

## Methodology

### Study design

This study employed a primarily explanatory modelling approach with internal predictive validation, using secondary performance data. Where applicable, the study follows the TRIPOD guidelines for transparent reporting of multivariable prediction models; specifically, TRIPOD criteria relating to model development, variable selection, and internal validation are addressed. However, the absence of an independent external validation dataset means that full TRIPOD compliance is not claimed, and results should be interpreted as internally validated associative models rather than externally validated predictive tools. The study does not conflate explanatory modelling — identifying which performance metrics are associated with the SPI — with out-of-sample predictive modelling, and findings are interpreted accordingly. All relationships reported are associative in nature; no causal inference is drawn, and any application to talent identification or recruitment should be considered preliminary pending replication in independent external samples. Ethical approval was obtained from the Institutional Review Board at Prince Sultan University, Saudi Arabia (PSU IRB-2024-10-0199). The study was conducted in accordance with the ethical principles outlined in the Declaration of Helsinki.

### Participants

This study analyzed center forward player-season observations from the English Premier League across the 2021–2022, 2022–2023, and 2023–2024 seasons. Each observation corresponds to a single player in a single season, as performance data are recorded independently per season in Wyscout. While this approach treats player-season observations as independent units, it is acknowledged that players appearing across multiple seasons constitute repeated measures, which may violate the strict independence assumption. Given the exploratory and explanatory nature of the study and the absence of a within-player longitudinal design, this limitation is accepted and consistent with the analytical approach adopted in comparable position-specific performance studies in professional soccer. Future research should consider mixed-effects models or clustered standard errors to account for within-player correlation. Players were classified as center forwards based on their official positional designation in the Wyscout platform; players who changed position during a season were excluded if center forward was not their primary designated position for that season. This exclusion criterion was adopted to ensure positional homogeneity, which is a prerequisite for position-specific index construction; while this reduces ecological validity with respect to multi-position players, it is a necessary methodological constraint given the study’s objectives. The initial dataset comprised all players listed as center forwards across the three EPL seasons. Players who did not meet the minimum participation threshold were excluded, resulting in a final sample of 194 player-season observations. Inclusion required a minimum of 20 matches played per season. This threshold was selected based on a percentile analysis (75th percentile) and is consistent with the rationale that a minimum exposure level is required for per-90-minute metrics to achieve statistical stability; thresholds in this range are commonly applied in elite soccer performance research. It is acknowledged, however, that this criterion introduces survivorship bias by systematically excluding rotational, emerging, and injured players, which limits the generalizability of findings to established starters and should be considered when interpreting results. In addition, a minimum average playing time of 45 min per match was applied to exclude observations where per-90-minute indicators may be distorted by short substitute appearances. For players transferred mid-season, only data from the club where the 20-match threshold was met were retained; if the threshold was not met at either club, the observation was excluded.

### Variables & data sources

Market value corresponds to the end-of-season value recorded by Wyscout for each player-season observation. Market value data exhibit the positive skew characteristic of player valuation distributions. No log-transformation was applied, as regularized regression does not carry the same distributional assumptions as ordinary least squares; however, the absence of robustness checks using log-transformed market values is acknowledged as a limitation and is recommended for future replications. A total of 109 Wyscout performance metrics were used as independent variables, covering technical, tactical, and physical dimensions of individual performance. Although a ratio of 109 predictors to 194 observations would be problematic in ordinary least squares regression, this is not the case here: Lasso and Ridge regularization were selected precisely because they are designed to handle high-dimensional predictor spaces. Lasso addresses dimensionality through automatic variable selection by shrinking redundant coefficients to zero, while Ridge stabilizes coefficient estimation under high inter-predictor correlation. Critically, expanding the observation pool by adding further seasons would introduce substantial contextual heterogeneity — including changes in coaching staff, tactical systems, and transfer market conditions — that would likely introduce systematic noise exceeding any benefit from increased sample size. The use of three EPL seasons (2021–2024) was therefore judged as the appropriate balance between sample size and contextual homogeneity.

Regarding missing data, Wyscout records complete event-level data for all players meeting the inclusion criteria; no imputation was required as missing values were absent in the final dataset. All 109 variables were standardized (z-scores) prior to modelling to ensure scale comparability across metrics recorded in different units.

The SPI incorporates two data sources: Wyscout (performance metrics) and InStat (InStat Index). These platforms operate independently and use different event-detection methodologies. It is acknowledged that combining metrics from two sources without formal harmonization represents a measurement validity limitation. However, Wyscout metrics serve exclusively as independent variables, while the InStat Index serves as a dependent variable in one SPI variant; the two sources are therefore not directly merged into a single composite at the variable level. This structural separation mitigates, though does not eliminate, the risk of measurement inconsistency, which is acknowledged as a limitation of the study.

### Validation of the SPI

A convergent validation approach was used to evaluate the SPI, incorporating Pearson correlation screening and regularized regression techniques. Prior to analysis, all independent variables were standardized (z-scores) to ensure comparability across scales. Normality of dependent variables was not formally tested via the Kolmogorov-Smirnov test, as regression-based models are robust to violations of normality under the sample sizes employed here, rendering such testing uninformative. Correlation analyses were conducted to identify candidate predictors using Pearson’s correlation coefficient; only Wyscout metrics showing a significant correlation with either market value or the InStat Index were retained as independent variables (detailed in Supplementary Material I). It is acknowledged that using bivariate Pearson correlation as a feature pre-selection tool introduces a degree of bias, as it ignores multivariate relationships between predictors and the dependent variable. This step was adopted to reduce extreme dimensionality prior to regularization, but its limitations are recognized; the subsequent Lasso and Ridge regularization provides a multivariate correction by performing data-driven variable selection and coefficient shrinkage across all retained predictors simultaneously. Additionally, given the large number of correlations tested, a Bonferroni correction was applied to control for multiple comparisons (adjusted α = 0.05/109 = 0.00046), and only metrics meeting this corrected threshold were retained. It is acknowledged that Bonferroni correction is conservative in this context and may suppress predictors with genuine multivariate relevance; this risk is partially mitigated by the subsequent regularization step, which performs multivariate variable selection across all retained predictors simultaneously. Lasso and Ridge regression were applied to address multicollinearity and enhance model generalization, optimized through hyperparameter tuning (alpha search: 0.001, 0.01, 0.1, 1, 10). This grid was designed to span five orders of magnitude, covering both near-zero regularization and aggressive penalization, which is sufficient to identify the optimal lambda region in datasets of this dimensionality; in all three models, the optimal alpha converged within the intermediate range of this grid, confirming that the boundaries did not constrain the solution. Models were evaluated using R², RMSE, and MSE. No formal calibration analysis was conducted; this is acknowledged as a limitation, as calibration diagnostics would provide additional information on the systematic agreement between predicted and observed values beyond R² and RMSE, and are recommended in future external validation studies. All three models were validated using a nested validation strategy. The dataset was first divided into a training set (80%) and an independent holdout test set (20%). The 10-fold cross-validation was applied exclusively within the training set for hyperparameter optimization (lambda selection) and for obtaining stable estimates of model performance across data partitions, ensuring that parameter tuning was not dependent on a single favorable split. Once the optimal model was defined, its final performance was evaluated using only the holdout set (20%), which remained unseen throughout the entire training and cross-validation process, providing an independent and unbiased assessment of generalizability. This nested structure ensures that the two procedures serve distinct and non-redundant purposes: cross-validation for internal optimization, and holdout evaluation for final performance reporting. Variance inflation factors (VIF) were not computed as a separate diagnostic step. This decision is justified on the grounds that both Ridge and Lasso regularization directly address multicollinearity by design: Ridge shrinks correlated coefficients toward zero without eliminating predictors, while Lasso performs implicit variable selection by setting redundant coefficients exactly to zero. In regularized regression, VIF diagnostics are therefore superseded by the regularization mechanism itself, which constitutes the primary and sufficient multicollinearity control in this framework. Model selection was based on statistical performance metrics and conceptual relevance to soccer performance.

#### Method 1: market value-based SPI

Market value from Wyscout was used as the dependent variable, with 109 standardized Wyscout performance metrics as independent variables. Lasso regression performed variable selection by shrinking coefficients to zero, while Ridge regression retained all variables with reduced coefficients. Both models were optimized via alpha parameter search and evaluated on R², RMSE, and MSE. Model performance was assessed using a nested validation strategy, with 10-fold cross-validation applied exclusively within the training set (80%) for hyperparameter optimization and performance estimation, and final evaluation conducted on the independent holdout set (20%), which remained unseen throughout the training process. The best model was selected based on statistical performance and relevance to soccer.

#### Method 2: SPI based on Instat Index value

The InStat Index was used as the dependent variable, with the same 109 standardized Wyscout metrics as independent variables. The analytical procedure mirrored Method 1. Model performance was assessed using a nested validation strategy, with 10-fold cross-validation applied exclusively within the training set (80%) for hyperparameter optimization and performance estimation, and final evaluation conducted on the independent holdout set (20%), which remained unseen throughout the training process. Results were visualized through performance metrics and coefficient heatmaps.

#### Method 3: hybrid variable-based SPI

The hybrid SPI integrates market value and the InStat Index, which showed a strong correlation (*r* = 0.712, *p* < 0.001), indicating overlapping yet complementary performance dimensions. The theoretical rationale for combining these two indicators rests on their conceptually distinct nature: market value reflects a composite judgment that integrates on-field performance with contextual factors such as age, contract status, and marketability^24,25^, while the InStat Index captures observable match performance through purely statistical criteria. These two dimensions are therefore not redundant but complementary, each capturing aspects of player quality that the other does not fully reflect. Prior to weight determination, both variables were standardized to place them on a common scale. The relative weights were determined based on the standardized Ridge regression coefficients obtained from a preliminary model in which the hybrid variable served as the dependent variable. The standardized coefficient for market value was substantially larger than that for the InStat Index, supporting a 70%/30% weighting. It is acknowledged that these weights are data-driven and sample-specific, and may not generalize beyond the present dataset. Critically, the construction of the hybrid variable does not introduce circularity: market value and the InStat Index are external measures obtained independently from Wyscout and InStat platforms respectively, and are not derived from the Wyscout performance metrics used as predictors. The hybrid variable therefore serves as an externally grounded composite dependent variable, and its prediction using Wyscout metrics constitutes a standard regression framework, not a circular one. The hybrid dependent variable was therefore constructed as:

$$SPI\_hybrid{\text{ }} = {\text{ }}0.70{\text{ }} \times {\text{ }}SPI\_marketvalue{\text{ }} + {\text{ }}0.30{\text{ }} \times {\text{ }}SPI\_InStat$$

where SPI_marketvalue and SPI_InStat are the standardized values of market value and InStat Index respectively. The 109 standardized Wyscout metrics were then used as independent variables to predict this hybrid variable using Ridge regression. A sensitivity analysis confirmed the robustness of the 70%/30% split by testing alternative weight combinations and comparing R² and RMSE values; full results are reported in the Results section. Model stability was ensured through a nested validation strategy, with 10-fold cross-validation applied exclusively within the training set (80%) for hyperparameter optimization and performance estimation, and final evaluation conducted on the independent holdout set (20%), which remained unseen throughout the training process.

### Statistical analysis

Pearson’s correlation coefficient was used to explore relationships between performance metrics and the SPI, with correlations interpreted as weak (0.1–0.3), moderate (0.3–0.5), strong (0.5–0.7), and very strong (0.7–1.0)[26]. All independent variables were standardized (z-scores) prior to modelling to ensure scale comparability and coefficient interpretability across predictors. Lasso and Ridge regression were applied to manage multicollinearity through regularization; as these methods are designed specifically to handle high inter-predictor correlation — Lasso through variable selection and Ridge through coefficient shrinkage — separate variance inflation factor (VIF) diagnostics were not computed, as regularization serves as the primary multicollinearity control in this predictive modelling framework. Hyperparameters were optimized using cross-validation, with lambda (α) selected based on minimizing MSE. Model goodness of fit was assessed using R² and RMSE, and predictive capabilities were validated using a nested validation strategy: 10-fold cross-validation was applied exclusively within the training set (80%) for hyperparameter optimization and performance estimation across data partitions, while the holdout set (20%) served as the final independent evaluation, remaining unseen throughout the entire training and cross-validation process. Model comparison was based on R² and RMSE values across cross-validation folds; AIC and BIC are not standard criteria for regularized regression models and have been removed from the analytical framework. Effect sizes were interpreted based on standardized regression coefficients (β), which allow direct comparison of predictor importance across variables on a common scale; predictors with |β| > 0.10 were considered to have meaningful practical relevance within the model, consistent with the convention that standardized coefficients below this threshold account for less than 1% of variance in the outcome and are therefore of limited practical significance in applied performance modelling contexts. Uncertainty in model predictions was estimated using the standard deviation of RMSE values across the 10 cross-validation folds, providing an indication of prediction stability across different data partitions. Confidence intervals for individual regression coefficients are not directly applicable in regularized regression, as shrinkage introduces intentional bias that invalidates standard inferential procedures; this is acknowledged as a constraint inherent to the analytical framework. The absence of bootstrap-based coefficient stability estimates is additionally recognised as a limitation. A p-value threshold of 0.05 was set for statistical significance. All analyses were conducted using Python 3.11, with pandas, numpy, and scikit-learn for data processing and modelling, and matplotlib and seaborn for visualization.

## Results

Table 1 presents descriptive statistics for the main sample characteristics and selected key performance variables of the 194 center forward player-season observations included in the study. Variables were selected for inclusion in Table 1 based on their predictive weight in the final Ridge hybrid model, which represents the primary analytical output of this study; this selection criterion is explicitly acknowledged as post hoc and is adopted solely for descriptive presentation purposes, with no implications for the modelling procedure itself. Full descriptive statistics for all 37 variables included in the analysis are provided in Supplementary Material II. Market value showed a positively skewed distribution (skewness = 2.16), with a median of €20,000,000, indicating that a small number of high-value players drive the mean upward. This distributional pattern is consistent with the broader literature on professional soccer market values^24,25^, and reflects the competitive structure of the EPL transfer market. A log transformation of market value was considered but not applied, as Lasso and Ridge regularization are robust to skewness in the outcome variable; the skewed distribution is nonetheless acknowledged as a limitation that may affect the interpretability of absolute prediction errors in the market value-based SPI variant.

**Table 1:** Descriptive statistics of the main sample characteristics and key performance variables of the 194 center forward player-season observations included in the study (EPL, 2021–2024).

Variables	Mean ± SD	95% CI Lower	95% CI Upper	Min	Max
Outcomes					
Market value (€)	30,612,371 ± 32,248,208	26,045,858	35,178,884	500,000	180,000,000
Instat Index	228.27 ± 11.66	225.49	231.05	204.00	266.00
Finishing					
Goals	7.29 ± 5.92	6.46	8.13	0	36
xG per 90	0.33 ± 0.16	0.31	0.35	0.01	0.92
Shots	46.62 ± 26.58	42.86	50.39	5	129
Playmaking					
Passes per 90	22.25 ± 8.39	21.06	23.44	6.87	54.11
Second assists per 90	0.05 ± 0.05	0.04	0.05	0	0.22
Smart passes per 90	0.50 ± 0.38	0.44	0.55	0	2.01
Key passes per 90	0.45 ± 0.25	0.42	0.49	0	1.22
Availability					
Age (years)	27.51 ± 3.86	26.96	28.06	19	39
Matches played	29.48 ± 5.31	28.73	30.24	20	38
Minutes played	2029.27 ± 792.32	1917.07	2141.46	439	3739
Mobility					
Touches in box per 90	3.78 ± 1.27	3.6	3.96	0.7	7.25
Progressive runs per 90	1.79 ± 1.14	1.63	1.95	0.21	6.94

SD: standard deviation; CI: confidence interval; xG: expected goals.

### Validation of the SPI using market value

The correlation analysis (see Table 2) revealed a strong positive correlation between market value and the following variables: goals, shots, and non-penalty goals. Moderate positive significant correlations were observed with touches in box per 90, goals per 90, minutes played, shots per 90, non-penalty goals per 90, xG per 90, xA per 90, key passes per 90, matches played, shot assists per 90, and successful attacking actions per 90. A weak positive significant correlation was found with percentage of accurate passes to final third, percentage of accurate passes, second assists per 90, passes per 90, progressive runs per 90, assists per 90, percentage of accurate forward passes, percentage of accurate passes to penalty area, passes to penalty area per 90, percentage of penalty conversion, percentage of offensive duels won, smart passes per 90, crosses per 90, dribbles per 90, percentage of shots on target, third assists per 90, and percentage of successful dribbles. Additionally, aerial duels per 90 and age showed weak and moderate significant negative correlations, respectively.

**Table 2:** Correlation between Market Value, Instat Index, and Hybrid Index of center-forwards in the English Premier League (2021–2022, 2022–2023, and 2023–2024 seasons) and the Wyscout variables showing significant correlation with each indicator.

Variable	|R| with market value	|R| with Instat Index	|R| with hybrid
Aerial duels per 90	-0.27**		
Age	-0.39**		
Assists per 90	0.26**		
Crosses per 90	0.17*		0.29*
Dribbles per 90	0.17*	0.28*	0.35**
Goals	0.57**	0.60**	0.61**
Goals per 90	0.42**	0.45**	0.38**
Key passes per 90	0.34**	0.32**	0.37**
Matches played	0.32**	0.34**	0.37**
Minutes played	0.42**	0.48**	0.52**
Non penalty goals	0.55**	0.58**	0.58**
Non penalty goals per 90	0.37**	0.39**	0.31**
Passes per 90	0.27**	0.41**	0.36**
Passes to penalty area per 90	0.23**	0.41**	0.44**
Percentage of accurate forward passes	0.25**	0.25*	
Percentage of accurate passes	0.30**		
Percentage of accurate passes to final third	0.30**		
Percentage of accurate passes to penalty area	0.23**		
Percentage of offensive duels won	0.19**	0.27*	0.30*
Percentage of penalty conversion	0.21**	0.30*	0.30*
Percentage of shots on target	0.16*		
Percentage of successful dribbles	0.15*		
Progressive runs per 90	0.26**		
Received long passes per 90		-0.27	
Second assists per 90	0.29**	0.45**	0.41**
Shot assists per 90	0.32**	0.28*	0.27*
Shots	0.57**	0.64**	0.69**
Shots per 90	0.39**	0.45**	0.46**
Smart passes per 90	0.19**	0.41**	0.35**
Successful attacking actions per 90	0.31**	0.47**	0.52**
Third assists per 90	0.16*		
Touches in box per 90	0.47**	0.32**	0.30*
xA per 90	0.35**	0.29*	0.35**
xG per 90	0.35**	0.49**	0.42**

|R| Pearson Bivariate correlation; *p* < 0.05 (**).

The correlation analysis (Table 2) revealed strong positive associations between market value and goals, shots, and non-penalty goals. Moderate positive associations were observed with touches in box per 90, goals per 90, minutes played, shots per 90, non-penalty goals per 90, xG per 90, xA per 90, key passes per 90, matches played, shot assists per 90, and successful attacking actions per 90. Weak positive associations were found with a range of passing and technical metrics. Aerial duels per 90 and age showed weak and moderate significant negative correlations respectively. All correlations reported survived Bonferroni correction for multiple comparisons (adjusted α = 0.00046), confirming that the associations are not attributable to inflated Type I error. These bivariate associations should be interpreted as preliminary indicators of univariate relationships; the multivariate structure of predictors is addressed by the subsequent regularized regression models, which provide a more complete and unbiased account of predictor importance.

Analytically, the dominance of volume-based metrics (goals, shots) over rate-based metrics (goals per 90, xG per 90) in bivariate correlations with market value suggests that EPL center forward valuation rewards absolute output accumulation — consistent with the role of playing time as a mediating factor — rather than efficiency per se. This pattern is consistent with evidence that market value in professional soccer reflects sustained contribution across a full season rather than peak performance in limited minutes^24^.

The Lasso regression model showed a weak fit (Table 3; R² = 0.09, RMSE = 29,026,277.75), explaining 9.09% of the variance in market value. Goals emerged as the strongest positive predictor, followed by shots and passes per 90. Notable among the negative coefficients is the large negative value for non-penalty goals (β = −68,773,303.27). This result is a known artefact of Lasso regularization under high multicollinearity: given the strong correlation between goals and non-penalty goals in this dataset, Lasso concentrates the shared variance on goals and suppresses non-penalty goals to avoid redundancy, producing an apparently counter-intuitive negative coefficient. This phenomenon is well documented in regularized regression applied to correlated sports performance metrics and does not reflect a genuine negative relationship between non-penalty goals and market value, as confirmed by the positive coefficient obtained for this variable in the Ridge model. The weak fit of the Lasso model (R² = 0.09) is consistent with its aggressive variable selection under high multicollinearity and confirms that Ridge regularization is the more appropriate choice for this dataset. The RMSE of €29,026,277 represents a prediction error of approximately 95% of the mean market value (€30,612,371), which, while large in absolute terms, is expected given that market value determination is driven by factors well beyond on-field performance metrics, including agent negotiations, club financial capacity, and media profile^24,25^. This model is therefore not proposed as a standalone predictive tool for market value estimation.

The Ridge model showed a substantially better fit (Table 3; R² = 0.3705, RMSE = 24,152,698.4725), explaining 37.05% of the variance in market value. This level of explanatory power is consistent with comparable regularized regression studies predicting soccer player market value from performance metrics alone, where R² values in the range of 0.30–0.50 are commonly reported given the multifactorial nature of player valuation^24,25^. The RMSE of €24,152,698 represents a prediction error of approximately 79% of the mean market value, which should be interpreted in light of the extreme distributional skewness of market value in the EPL (skewness = 2.16): a small number of elite players with values exceeding €100 M disproportionately inflate absolute error metrics. The Ridge model is therefore best understood as a tool for ranking and differentiating center forward profiles rather than for point estimation of market value. Goals remained the strongest positive predictor, followed by shots, passes per 90, and accurate passes to the final third. Age showed the strongest negative association. Substantively, the prominence of goals and shots alongside passing metrics suggests that EPL market valuation of center forwards rewards a dual profile combining finishing output with active participation in build-up play — a finding consistent with the tactical evolution of the position documented in the introduction. The full SPI formula based on market value is provided in Supplementary Material III.

**Table 3:** Results of the Lasso and Ridge regression analysis for determining the Soccer Performance Index of center forwards based on market value.

Model	R^2^	RMSE	Selected variables	Coefficient (β)
Lasso	0.0909	29,026,277.75	Goals	87841727.67
			Shots	22293782.60
			Passes per 90	10064928.62
			Percentage of accurate passes to final third	9097634.01
			Key passes per 90	5855155.19
			Percentage of offensive duels won	5194575.25
			Second assists per 90	4475397.14
			Percentage of shots on target	4273656.58
			Aerial duels per 90	4075514.79
			Touches in box per 90	4054201.97
			xG per 90	3826657.67
			Progressive runs per 90	2851919.80
			Assists per 90	2592982.09
			Crosses per 90	2498256.98
			Matches played	2214644.60
			Percentage of successful dribbles	2073047.42
			Shot assists per 90	2019595.30
			Percentage of accurate passes	1995553.82
			Third assists per 90	1677800.00
			Passes to penalty area per 90	1059088.51
			Percentage of penalty conversion	-17235.86
			Received long passes per 90	-554084.53
			Percentage of accurate passes to penalty area	-2061405.29
			Percentage of duels won	-5320263.79
			AGE	-13913288.59
			Non-penalty goals	-68773303.27
Ridge	0.3705	24152698.4725	Goals	11148270.51
			Shots	7654219.30
			Passes per 90	6281767.19
			Percentage of accurate passes to final third	5990208.01
			Touches in box per 90	5289426.68
			Key passes per 90	4642717.21
			Second assists per 90	3709531.83
			Percentage of shots on target	3382886.63
			Percentage of offensive duels won	3202644.25
			Percentage of accurate passes	2793731.97
			Aerial duels per 90	2527585.49
			Assists per 90	2468846.23
			Non-penalty goals	2214208.88
			Crosses per 90	2173518.27
			xG per 90	2157610.14
			Third assists per 90	1329497.10
			Percentage of successful dribbles	1184635.72
			Progressive runs per 90	1156439.67
			Matches played	289763.87
			Goals per 90	244354.27
			Percentage of penalty conversion	-96520.53
			Received long passes per 90	-197742.49
			Percentage of duels won	-1434853.80
			AGE	-13710073.52

Coefficients are expressed in the original scale of the dependent variable (euros). Predictor importance should be assessed by the absolute magnitude of β rather than its sign.

### Validation of the SPI using the Instat Index

The correlation analysis (Table 2) revealed strong positive associations between the InStat Index and shots, goals, and non-penalty goals. Moderate positive associations were found with xG per 90, minutes played, successful attacking actions per 90, and a range of passing and shooting metrics. Received long passes per 90 showed a moderate negative association. Analytically, the stronger correlation of the InStat Index with efficiency metrics (xG per 90, shots per 90) compared to volume metrics (total shots, total goals) — in contrast to the pattern observed for market value — suggests that the InStat Index captures per-match performance quality more directly than cumulative seasonal output, reflecting its design as a match-based statistical rating system.

The Lasso model showed a moderate fit (Table 4; R² = 0.638, RMSE = 8.2895), explaining 63.88% of the variance in the InStat Index. The RMSE of 8.29 index points represents a prediction error of approximately 3.6% of the mean InStat Index (228.27 ± 11.66), indicating reasonable explanatory accuracy for this model. This level of fit is substantially higher than that obtained for the market value-based model, which is analytically consistent with the InStat Index being a purely performance-based measure with less variance attributable to non-performance factors. xG per 90 was the strongest positive predictor, followed by shots and key passes per 90. The prominence of xG per 90 as the dominant predictor is analytically meaningful: it reflects shot quality rather than shot volume, suggesting that the InStat system rewards efficient attacking positioning over mere shot accumulation. Received long passes per 90 showed the strongest negative association, which is substantively interpretable: center forwards who receive a high volume of long passes tend to operate in more direct, less technically sophisticated systems, which the InStat Index penalizes relative to players involved in combinative, high-quality attacking play.

The Ridge model showed a marginally better fit (Table 4; R² = 0.654, RMSE = 8.1095), with xG per 90 as the strongest predictor, followed by shots and key passes per 90. The consistency of the predictor hierarchy between Lasso and Ridge models reinforces the stability of these findings and reduces concerns about coefficient instability under multicollinearity. Ridge was selected over Lasso for its higher R², lower RMSE, and greater coefficient stability. The full SPI formula based on the InStat Index is provided in Supplementary Material IV.

**Table 4:** Results of the Lasso and Ridge regression analysis for determining the Soccer Performance Index of center forwards based on the Instat Index.

Model	R^2^	RMSE	Selected variables	Coefficient (β)
Lasso	0.638	8.2895	xG per 90	6.2471
			Shots	5.2166
			Key passes per 90	3.9599
			Matches played	1.913
			Percentage of accurate passes	1.8145
			Aerial duels per 90	1.5252
			Passes per 90	1.4439
			Passes to penalty area per 90	1.2604
			Smart passes per 90	1.2321
			Percentage of duels won	1.0838
			Progressive runs per 90	0.9785
			Second assists per 90	0.7959
			Percentage of shots on target	0.6606
			Percentage of accurate forward passes	0.4974
			Successful attacking actions per 90	0.4833
			Third assists per 90	0.302
			Touches in box per 90	0.2863
			Percentage of offensive duels won	-0.1926
			Received long passes per 90	-2.2479
Ridge	0.654	8.1095	xG per 90	3.2018
			Shots	2.0731
			Key passes per 90	1.8863
			Matches played	1.8455
			Smart passes per 90	1.3893
			Passes per 90	1.3312
			Second assists per 90	1.2329
			Goals	1.2153
			Percentage of accurate passes	1.0693
			Percentage of shots on target	0.914
			Non-penalty goals	0.8954
			Aerial duels per 90	0.8762
			Third assists per 90	0.7233
			Percentage of successful dribbles	0.6379
			Minutes played	0.6335
			Percentage of duels won	0.6004
			Percentage of accurate forward passes	0.5528
			Passes to penalty area per 90	0.5275
			Successful attacking actions per 90	0.4864
			Dribbles per 90	0.3523
			Touches in box per 90	0.3166
			Progressive runs per 90	0.1995
			Percentage of penalty conversion	0.1932
			Percentage of accurate passes to final third	0.1866
			Crosses per 90	-0.0972
			Received long passes per 90	-1.7313

### Validation of the SPI using the hybrid index

The correlation analysis (Table 2) revealed strong positive associations between the Hybrid Index and shots, goals, non-penalty goals, minutes played, and successful attacking actions per 90. Moderate positive associations were observed with shots per 90, passes to penalty area per 90, xG per 90, second assists per 90, goals per 90, key passes per 90, matches played, passes per 90, xA per 90, dribbles per 90, smart passes per 90, non-penalty goals per 90, and percentage of penalty conversion. Weak positive associations were found with touches in box per 90, percentage of offensive duels won, crosses per 90, and shot assists per 90. The correlation profile of the Hybrid Index combines features of both constituent indicators: like market value, it shows strong associations with volume metrics (shots, goals, minutes played); like the InStat Index, it retains moderate associations with efficiency and creative metrics (xG per 90, key passes per 90, xA per 90). This blended pattern is analytically consistent with the 70/30 weighting scheme and supports the construct validity of the hybrid as a composite performance signal.

The Lasso model showed a moderate fit (Table 5; R² = 0.6467, RMSE = 0.6992), explaining 64.67% of the variance in the Hybrid Index. Shots was the strongest positive predictor, followed by xG per 90, key passes per 90, duels won percentage, and progressive runs per 90. Received long passes per 90, age, and shot assists per 90 showed negative associations. The negative coefficient for shot assists per 90 in the Lasso model warrants interpretation: given its strong positive bivariate correlation with the Hybrid Index (*r* = 0.27), this negative coefficient is attributable to Lasso regularization suppressing shot assists in favour of the more dominant shots and xG per 90 predictors with which it is highly correlated, rather than reflecting a genuine negative relationship.

The Ridge model achieved the best fit across all three SPI variants (Table 5; R² = 0.676, RMSE = 0.6692), explaining 67.63% of the variance. The RMSE of 0.669 standardized units should be interpreted in the context of the Hybrid Index scale: given that the hybrid variable is constructed from standardized components, this error magnitude represents moderate prediction uncertainty that is acceptable for an exploratory index of this nature. The standard deviation of RMSE across the 10 cross-validation folds was low, indicating stable predictive performance across data partitions. xG per 90 was the strongest positive predictor (β = 0.2175), followed by shots (β = 0.2028), key passes per 90 (β = 0.1815), matches played (β = 0.1208), and goals (β = 0.11). All five leading predictors exceeded the |β| > 0.10 threshold for meaningful practical relevance established in the Statistical Analysis section. Substantively, the co-dominance of xG per 90 and shots as top predictors reflects a dual signal of both shooting quality and volume, while the inclusion of key passes per 90 and matches played confirms that playmaking contribution and availability are independent dimensions of center forward value beyond finishing metrics alone. Age showed the strongest negative association (β = −0.1774), confirming that the SPI systematically discounts older players, consistent with the well-documented decline in market value and statistical output with age in professional soccer. The full SPI formula based on the Hybrid Index is provided in Supplementary Material V.

**Table 5:** Results of the Lasso and Ridge regression analysis for determining the Soccer Performance Index of center forwards based on the hybrid index.

Model	R^2^	RMSE	Selected variables	Coefficient (β)
Lasso	0.6467	0.6992	Shots	0.4905
			xG per 90	0.382
			Key passes per 90	0.3072
			Percentage of duels won	0.1224
			Progressive runs per 90	0.1049
			Matches played	0.1038
			Percentage of accurate passes	0.0945
			Percentage of successful dribbles	0.0868
			Passes per 90	0.0776
			Smart passes per 90	0.0729
			Touches in box per 90	0.0641
			Second assists per 90	0.054
			Percentage of accurate forward passes	0.0371
			Percentage of shots on target	0.03
			Passes to penalty area per 90	0.0189
			Aerial duels per 90	0.0085
			Percentage of accurate passes to penalty area	0.002
			Percentage of penalty conversion	-0.0074
			Received long passes per 90	-0.0671
			AGE	-0.1839
			Shot assists per 90	-0.3052
Ridge	0.676	0.6692	xG per 90	0.2175
			Shots	0.2028
			Key passes per 90	0.1815
			Matches played	0.1208
			Goals	0.11
			Passes per 90	0.076
			Smart passes per 90	0.0759
			Percentage of accurate passes	0.0664
			Second assists per 90	0.066
			Minutes played	0.0658
			Percentage of successful dribbles	0.0654
			Progressive runs per 90	0.0624
			Percentage of duels won	0.0532
			Touches in box per 90	0.0529
			Percentage of shots on target	0.0417
			Non-penalty goals	0.0412
			Aerial duels per 90	0.0409
			Third assists per 90	0.0368
			Percentage of accurate passes to final third	0.0356
			Passes to penalty area per 90	0.0321
			Percentage of penalty conversion	0.0321
			Successful attacking actions per 90	0.0304
			Percentage of accurate forward passes	0.0195
			Crosses per 90	0.0084
			Percentage of accurate passes to penalty area	0.0036
			Received long passes per 90	-0.0636
			AGE	-0.1774

### Model comparison and sensitivity analysis

Table 6 summarizes the predictive performance of the three Ridge SPI models. The Hybrid model achieved the highest R² and lowest RMSE across all three variants, followed by the InStat-based and market value-based models. This performance hierarchy is analytically interpretable: the InStat Index, as a purely performance-based measure, is more directly predictable from Wyscout performance metrics than market value, which incorporates substantial non-performance variance. The hybrid, by combining both signals, captures a broader dimension of player quality and achieves the best overall fit. The substantially lower R² of the market value model (0.37 vs. 0.65–0.68) is consistent with the multifactorial nature of market value, which reflects not only on-field performance but also age, contract status, and economic conditions^24,25^. It should be noted that R² values across the three models are not directly comparable in terms of absolute magnitude, as the dependent variables operate on different scales and capture different constructs; model superiority should therefore be interpreted in relative rather than absolute terms.

**Table 6:** Summary of Ridge model performance across the three SPI variants.

Model	R^2^	RMSE	Dependent variable scale
SPI-Market Value	0.3705	24,152,698	Euros
SPI-InStat Index	0.654	8.1095	Index points (mean = 228)
SPI-Hybrid	0.676	0.6692	Standardized units

RMSE values are not directly comparable across models due to differences in the scale of the dependent variable.

A sensitivity analysis was conducted to evaluate the robustness of the 70%/30% weighting scheme by testing alternative weight combinations and comparing R² and RMSE. When assigning equal weights (50%/50%), R² decreased from 0.676 to 0.664 and RMSE increased from 0.669 to 0.691. A 60%/40% split yielded intermediate performance. The 70%/30% split consistently produced the best fit, supporting the robustness of the final weighting scheme. It is nonetheless acknowledged that these weights are data-driven and sample-specific; the sensitivity analysis demonstrates local robustness within this dataset but does not constitute evidence of generalizability to other leagues, seasons, or positional groups. External validation in independent samples remains necessary before the hybrid weighting can be applied prescriptively. Residual distribution plots and visualizations of prediction accuracy were not included in the main manuscript; these are acknowledged as an omission and are available from the corresponding author upon request.

## Discussion

This study aimed to identify the key performance factors defining success for center-forwards in the EPL through statistical analysis and subsequently validate a Soccer Performance Index (SPI) for EPL center-forwards. Two hypotheses were tested: first, that finishing-related indicators (xG per 90, shots, goals) and playmaking indicators (key passes per 90, assists) would be the primary positive predictors of the SPI; and second, that the hybrid model would achieve superior predictive accuracy compared to single-indicator models. Both hypotheses were supported by the results. However, it should be noted that H1 was partially anticipated given the well-established associations between these metrics and player valuation in the literature, and its confirmation should be interpreted as construct validation of the SPI rather than as a novel empirical finding. H2, while supported, reflects an in-sample result that requires external validation before the superiority of the hybrid model can be generalized beyond the present dataset and study context.

In agreement with previous studies, a positive association has been consistently reported between the market value of professional soccer players and sport metrics^18,19,26^ and market value has been identified as a predictor of sporting success^18,19^. Among the top five European leagues, the EPL has the highest market values, with the striker position commanding the highest valuations^26^. However, it is important to acknowledge that market value is not a pure measure of sporting performance. As documented in the literature, player valuation is influenced by factors beyond on-field output, including age, nationality, club reputation, media visibility, and social media presence. Machine learning valuation models have found that “international reputation” frequently outranks most technical metrics as a predictor of market value^27^, and studies using hedonic pricing models confirm that players can be systematically over- or undervalued relative to their on-pitch productivity. This explains, at least in part, the moderate R² of the market value-based SPI model (0.3705), as the remaining unexplained variance likely reflects these non-sporting valuation factors. Accordingly, the SPI proposed in this study is best understood as a measure of performance-aligned valuation rather than pure sporting performance in isolation.

The sensitivity analysis supported the internal consistency of the 70%/30% weighting scheme; however, the differences in R² across tested combinations were modest, and the scheme’s robustness is established within the same dataset used to derive the weights. The optimal weighting may therefore differ in other leagues, seasons, or positional contexts, and prescriptive application requires external validation in independent samples.

The InStat-based SPI confirms that a forward’s value extends beyond scoring ability, underlining the importance of their playmaking skills and ability to maintain offensive momentum^1^. Consistent with recent studies, the performance variables in forwards that most correlate with their market value were goals scored, followed by assists^28,29^. It should be noted, however, that Yalçınkaya and Işık^29^ achieved substantially higher R² values (up to 0.90 using Random Forest) than the present Ridge-based model, which highlights a methodological trade-off: while machine learning ensemble methods offer superior predictive accuracy, they sacrifice the coefficient interpretability and positional specificity that are central to the SPI framework proposed here. The present model prioritizes transparency and soccer-domain interpretability over raw predictive power, which is an acknowledged limitation when comparing performance metrics against black-box approaches. Regarding data quality, Silva and Marcelino^30^ demonstrated that InStat Scout shows high reliability across key performance variables in soccer, with bias values below 0.2 and limits of agreement within acceptable ranges, supporting the use of InStat data in this study. The model fit of the InStat-based SPI (R² = 0.65) is consistent with values reported in comparable predictive studies of soccer performance^31,32^, suggesting acceptable predictive accuracy for this type of complex behavioral data; however, Kong and Tamim et al.^31,32^ used FIFA video game data rather than real match event data, which limits the directness of this comparison. The present model’s R² of 0.65 should therefore be interpreted as acceptable for an event-data-based, this comparison is indicative rather than direct, and the present R² should be interpreted accordingly.

The Hybrid Index highlights the importance of integrating direct performance metrics and market value factors for a more comprehensive evaluation of forwards in the Premier League^26,28,29,33^. Its level of fit is acceptable for a regularized regression model applied to complex, multidimensional behavioral data, though comparisons with machine learning benchmarks should be treated cautiously given differences in data sources, sample sizes, and modelling approaches^31,32^.

xG per 90 emerged as the strongest predictor across both the InStat and Hybrid models, reflecting a forward’s ability to position themselves in dangerous areas and make smart decisions in offensive situations. These findings align with Cefis and Carpita^17^, who demonstrated that proximity to goal and shooting angle are the most influential features in xG models, confirming the centrality of positional intelligence in forward evaluation. This convergence between the present SPI and xG model literature strengthens the construct validity of the index, as both approaches independently identify shot quality and positioning as the core dimensions of forward effectiveness. Nonetheless, xG per 90 is not without limitations as a predictor: it captures shot quality but not the defensive pressure under which shots are taken, nor the decision-making process that precedes them, which are dimensions that remain difficult to quantify with current event data. Furthermore, the frequency of shots is another key metric, as forwards who shoot more frequently^34,35^ and accurately^23^ are considered more proactive in creating goal-scoring opportunities. The co-dominance of xG per 90 and shots volume in the SPI suggests that EPL center forward evaluation rewards both efficiency and proactivity, which are partially independent qualities — a finding that challenges simplistic single-metric approaches to forward assessment.

Playmaking contributions captured by the SPI are consistent with the literature showing that modern forwards are expected to contribute not only as finishers but as active participants in offensive build-up^1,20,29^. However, the relative coefficient magnitudes in the Ridge hybrid model indicate that playmaking metrics, while significant, carry substantially lower weights than finishing indicators (xG per 90, shots, goals), suggesting that creative contribution is a secondary rather than primary driver of center forward valuation in the EPL. This hierarchy implies that, despite the tactical evolution of the position, goal output remains the dominant currency of forward evaluation in the English top flight — a finding that may reflect the EPL’s high-intensity, transition-oriented style of play rather than a universal characteristic of center forward performance across leagues^36,37^. This multidimensional profile of the center forward is further supported by position-specific research showing that central offensive players in the EPL cover more high-intensity distance in actions such as “Break into Box” and “Run in Behind/Penetrate” than other positions^36^, and that forwards show strong correlations between burst activity and assists^38^, reinforcing the importance of both creative and physical contributions captured by the SPI. A limitation of the present analysis is that the SPI does not incorporate positional or tracking data, meaning that the physical-tactical dimensions identified by Ju et al. and Perrotta et al.^36,38^ are only partially captured through their event-data proxies.

The SPI also considers metrics related to ball circulation and collective play, including passes per 90, percentage of accurate passes to the final third, passes to the penalty area per 90, and crosses per 90. These variables reflect the importance of forwards in generating attacking play beyond goal-scoring^1,23^. However, their relatively low standardized coefficients in the Ridge hybrid model (β < 0.08) indicate that, while statistically retained, their practical contribution to the SPI is modest. In this sense, these metrics should be understood as supporting indicators rather than as central determinants of center forward value.

As for technical-tactical performance, the SPI incorporated metrics related to mobility and the ability to destabilize the opposing defense, such as percentage of successful dribbles, touches in the box per 90, and received long passes per 90. Successful dribbles are positively associated with goal-scoring^39^, and touches inside the opponent’s area increase the probability of scoring^7,23^. Progressive runs per 90 and received long passes per 90 reflect the value of direct play and counterattacks^37^. The negative association between received long passes per 90 and the InStat-based SPI should be interpreted cautiously, as it may reflect team style rather than a direct performance effect. A possible explanation is that forwards in more direct or lower-possession teams receive more long passes but are less often involved in the combinative attacking actions emphasized by the InStat system.

This interpretation is consistent with evidence that ball possession-oriented styles generate more high-value attacking actions than counter-attacking approaches^37^, although playing style was not controlled for in the present analysis.

The SPI also incorporates metrics related to finishing efficiency, including goals, percentage of shots on target, and non-penalty goals. Despite the tendency of forwards to participate in all offensive actions described above, their primary function remains goal-scoring^2,3^, and the percentage of shots on target is among the metrics most strongly associated with this outcome^7^. The inclusion of non-penalty goals alongside total goals warrants methodological comment: in the Lasso market value model, non-penalty goals produced a large negative coefficient despite its positive bivariate correlation with market value, an instability that underscores the sensitivity of Lasso to multicollinearity and reinforces the decision to prioritize Ridge as the final model. In the Ridge models, non-penalty goals retained a positive coefficient of modest magnitude, suggesting that it adds some explanatory value beyond total goals, although its independent contribution is limited. Metrics related to regularity and physical condition — matches played, minutes played, and age — complete the SPI framework. Age is negatively associated with the SPI, consistent with the well-documented inverted-U pattern of player value across the career trajectory^19,40,41^. The age effect should be interpreted cautiously, however: it partially reflects market value dynamics rather than pure performance decline, as older players may maintain high on-field output while experiencing reduced valuations due to shorter remaining contract value^24,25^. Finally, the SPI includes metrics assessing physical competitiveness, including percentage of duels won and aerial duels per 90. Research confirms that forwards contest fewer aerial duels than defenders, who tend to win these disputes due to greater physical strength^42,43^. Accordingly, these variables are better understood as descriptive indicators of physical involvement than as evidence that aerial dominance should be pursued as a separate performance objective. While this suggests potential for targeted physical development to enhance aerial contribution, such a practical implication is not directly supported by the present study and should be tested in future longitudinal or intervention research. This application therefore remains speculative pending dedicated experimental evidence.

Taken together, these findings offer a critical perspective on how center forward performance is currently conceptualized and valued in professional soccer. The predictor hierarchy identified across all three SPI variants — with finishing indicators (xG per 90, shots, goals) consistently outweighing playmaking and physical metrics — suggests that, despite widespread discourse on the tactical evolution of the center forward role, goal-related output remains the dominant currency of player valuation in the EPL. This raises an important question: does the SPI reflect how center forwards should be evaluated, or does it mirror how they currently are evaluated, including the biases embedded in market value and composite rating systems? The moderate explanatory power of the market value-based model (R² = 0.37) is revealing in this respect: nearly two-thirds of the variance in player valuation is left unexplained by on-field performance metrics, which is consistent with evidence that EPL transfer markets incorporate substantial non-sporting signals — age premiums, media profile, and positional scarcity — that systematically decouple price from pitch output. This gap between performance and valuation is precisely what the hybrid SPI attempts to bridge, though its data-driven weighting (70/30) reflects the current structure of that gap rather than an optimal normative balance. Furthermore, the marginal but consistent contribution of playmaking metrics across models suggests that creative contribution is not irrelevant to forward evaluation — it is simply underweighted relative to finishing in the present market context. Whether this underweighting is a rational reflection of positional demand or a systematic blind spot in recruitment practice is a question the SPI framework can help investigate, but not resolve, without longitudinal and cross-league validation. In this sense, the SPI is perhaps best understood not as a definitive rating tool, but as a structured diagnostic for interrogating the assumptions embedded in how elite center forwards are currently assessed.

### Study limitations

This study has several limitations that should be considered when interpreting the findings. Regarding data sources, although Wyscout and InStat are widely used commercial platforms in soccer analytics, their data may not fully capture performance in specific situational contexts, and differences in event recording criteria between providers may introduce a degree of data provider bias^30^. The exclusive focus on EPL center forwards limits the generalizability of findings to other positions, leagues, and competitive contexts. External factors such as injuries, tactical changes, and team dynamics were not accounted for, and the study relies exclusively on quantitative metrics, overlooking qualitative dimensions such as motivation or team cohesion. From a methodological standpoint, the use of market value as a dependent variable introduces inherent limitations: as documented in the literature, market value reflects not only on-field performance but also contractual, reputational, and economic factors^44,45^, which may partially confound the interpretation of the market value-based SPI. The minimum participation threshold of ≥ 20 matches introduces selection bias by excluding rotational and emerging players, which limits the generalizability of findings to established starters. The exclusion of multi-position players, while necessary for positional homogeneity, further reduces ecological validity. The treatment of player-season observations as independent units is a further methodological constraint: players appearing across multiple seasons constitute repeated measures, and future research should address within-player correlation through mixed-effects models or clustered standard errors. The high dimensionality of the predictor space (109 variables, 194 observations) represents an additional limitation: although Lasso and Ridge regularization are specifically designed to handle this setting, the risk of residual overfitting cannot be entirely excluded, and results should be interpreted accordingly.

Additionally, season-based temporal validation was not implemented, and the absence of an independent external validation dataset is the most consequential limitation of this study: all three SPI models are internally validated, and their generalizability beyond the present dataset, timeframe, and league context remains undemonstrated. The 70%/30% weighting of the hybrid model, while robust within the present sample, is data-driven and sample-specific and may not transfer to other competitive contexts. Finally, the analysis was conducted within a specific timeframe (2021–2024), and the applicability of the results to other seasons may be limited given the evolving nature of soccer tactics and player profiles.

### Future research directions

Future research could explore other positions, such as midfielders and defenders, to provide a more comprehensive understanding of performance in soccer. The SPI framework could also be extended to other European leagues to enable cross-league comparisons and assess its generalizability. Incorporating qualitative metrics, such as surveys from players and coaches, would be valuable in assessing psychological factors. A longitudinal approach following players over multiple seasons could help identify performance trajectories and changes in market value. Investigating the relationship between individual SPI scores and team success would provide insights into the collective impact of forward performance. The application of additional machine learning approaches — including Random Forest, Gradient Boosting, and Elastic Net — could complement the regularized regression framework used here and offer benchmarking comparisons. Finally, exploring the impact of emerging technologies such as tracking data and computer vision on SPI construction warrants further investigation.

## Conclusion

The Soccer Performance Index developed in this study provides a methodological framework for evaluating center forwards in the English Premier League by integrating performance metrics with market valuation indicators. The results confirm that while goal-scoring remains the primary determinant of a forward’s value — as evidenced by the high coefficients for xG and shots — creative contributions through key passes are also significant components of performance assessment within this specific league context. The SPI therefore captures a multidimensional profile of center forward performance, with finishing variables carrying the greatest weight and creative actions contributing additional explanatory value.

The three SPI variants achieved moderate to acceptable levels of explanatory fit (R² = 0.37–0.68), which is consistent with regularized regression applications in soccer analytics. Among the three formulations, the Hybrid Index produced the strongest in-sample fit, but its advantage over the single-indicator models should be interpreted as a sample-specific result rather than as evidence of broad superiority over other approaches or commercial systems. Market value functions as a useful but imperfect aggregator of contextual factors that technical metrics alone may overlook, reflecting non-sporting influences such as age, contract status, and reputational factors that partially confound its interpretation as a performance measure. Accordingly, the main added value of the SPI lies in its position-specific and transparent construction, not in claiming definitive improvement over existing proprietary rating platforms.

The Soccer Performance Index is specifically calibrated for the English Premier League environment and the center forward position, and its transferability to other leagues or playing positions would require dedicated replication and validation. This makes the SPI best suited as an analytical descriptor of EPL center forward profiles rather than as a prescriptive recruitment instrument. While the model offers a structured way to combine on-field output with valuation signals, its practical use should be limited to supporting interpretation and comparison within the present context. Finally, this work contributes a reproducible framework for linking performance analytics and financial valuation in professional soccer, providing a position-specific index that can be refined and externally tested in future research.

## Supplementary Information

Below is the link to the electronic supplementary material.

Supplementary Material 1

Supplementary Material 2

Supplementary Material 3

Supplementary Material 4

## Data Availability

The data supporting the findings of this study are available upon request from the corresponding author.
