# Supplementary material for: A data-driven performance index for center forwards in the English Premier League
Source: Sci Rep. 2026 Jun 8;16:26001. doi: 10.1038/s41598-026-55681-9 (PMC13487182; doi:10.1038/s41598-026-55681-9)
Supplement: Supplementary file 1 [file 41598_2026_55681_MOESM1_ESM.docx]

**Supplementary Material II.** Descriptive Statistics of All Variables Included in the Analysis for the 194 Center Forward Player-Season Observations (EPL, 2021–2024).

| Variable | Mean ± SD | 95% CI Lower | 95% CI Upper | Min | Max |
| --- | --- | --- | --- | --- | --- |
| Accurate forward passes (%) | 63.84 ± 9.24 | 62.53 | 65.15 | 33.33 | 83.08 |
| Accurate passes to final third (%) | 66.50 ± 12.66 | 64.71 | 68.29 | 0 | 100 |
| Accurate passes (%) | 76.63 ± 5.86 | 75.8 | 77.46 | 60 | 89.47 |
| Accurate passes to penalty area (%) | 51.81 ± 12.78 | 50 | 53.62 | 0 | 80.95 |
| Aerial duels per 90 | 4.12 ± 2.94 | 3.7 | 4.53 | 0.36 | 13.83 |
| Age (years) | 27.51 ± 3.86 | 26.96 | 28.06 | 19 | 39 |
| Assists per 90 | 0.12 ± 0.10 | 0.11 | 0.13 | 0 | 0.48 |
| Crosses per 90 | 1.47 ± 1.13 | 1.31 | 1.63 | 0.06 | 4.75 |
| Dribbles per 90 | 3.47 ± 1.83 | 3.21 | 3.73 | 0.37 | 10.45 |
| Duels won (%) | 38.35 ± 5.81 | 37.52 | 39.17 | 24.67 | 55.26 |
| Goals | 7.29 ± 5.92 | 6.46 | 8.13 | 0 | 36 |
| Goals per 90 | 0.31 ± 0.19 | 0.28 | 0.34 | 0 | 1.08 |
| InStat Index | 228.27 ± 11.66 | 225.49 | 231.05 | 204 | 266 |
| Key passes per 90 | 0.45 ± 0.25 | 0.42 | 0.49 | 0 | 1.22 |
| Market value (€) | 30,612,371 ± 32,248,208 | 26045858 | 35178884 | 500000 | 180000000 |
| Matches played | 29.48 ± 5.31 | 28.73 | 30.24 | 20 | 38 |
| Minutes played | 2029.27 ± 792.32 | 1917.07 | 2141.46 | 439 | 3739 |
| Non-penalty goals | 6.53 ± 5.04 | 5.82 | 7.24 | 0 | 29 |
| Non-penalty goals per 90 | 0.28 ± 0.17 | 0.26 | 0.31 | 0 | 0.87 |
| Offensive duels won (%) | 35.57 ± 7.58 | 34.49 | 36.64 | 18.75 | 59.09 |
| Passes to penalty area per 90 | 1.81 ± 1.12 | 1.66 | 1.97 | 0.2 | 5.83 |
| Passes per 90 | 22.25 ± 8.39 | 21.06 | 23.44 | 6.87 | 54.11 |
| Penalty conversion (%) | 29.00 ± 43.46 | 22.85 | 35.15 | 0 | 0 |
| Progressive runs per 90 | 1.79 ± 1.14 | 1.63 | 1.95 | 0.21 | 6.94 |
| Received long passes per 90 | 1.68 ± 0.63 | 1.59 | 1.77 | 0.45 | 3.79 |
| Second assists per 90 | 0.05 ± 0.05 | 0.04 | 0.05 | 0 | 0.22 |
| Shot assists per 90 | 0.84 ± 0.44 | 0.78 | 0.9 | 0 | 2.29 |
| Shots | 46.62 ± 26.58 | 42.86 | 50.39 | 5 | 129 |
| Shots per 90 | 2.03 ± 0.67 | 1.94 | 2.13 | 0.23 | 3.99 |
| Smart passes per 90 | 0.50 ± 0.38 | 0.44 | 0.55 | 0 | 2.01 |
| Shots on target (%) | 41.37 ± 10.56 | 39.87 | 42.87 | 0 | 70.83 |
| Successful attacking actions per 90 | 3.30 ± 1.45 | 3.1 | 3.5 | 0.78 | 8.89 |
| Successful dribbles (%) | 51.01 ± 9.47 | 49.67 | 52.35 | 20 | 83.33 |
| Third assists per 90 | 0.03 ± 0.04 | 0.02 | 0.03 | 0 | 0.16 |
| Touches in box per 90 | 3.78 ± 1.27 | 3.6 | 3.96 | 0.7 | 7.25 |
| xA per 90 | 0.11 ± 0.07 | 0.1 | 0.12 | 0 | 0.35 |
| xG per 90 | 0.33 ± 0.16 | 0.31 | 0.35 | 0.01 | 0.92 |

Legend: SD: standard deviation; CI: confidence interval; xA: expected assist; xG: expected goals.
