# Supplementary material for: A data-driven performance index for center forwards in the English Premier League
Source: Sci Rep. 2026 Jun 8;16:26001. doi: 10.1038/s41598-026-55681-9 (PMC13487182; doi:10.1038/s41598-026-55681-9)
Supplement: Supplementary file 2 [file 41598_2026_55681_MOESM2_ESM.docx]

Supplementary Material III: SPI formula — Market Value

SPI = (11,148,270.51 × Goals) + (7,654,219.3 × Shots) + (6,281,767.188 × Passes per 90) + (5,990,208.011 × Percentage of accurate passes to final third) + (5,289,426.684 × Touches in box per 90) + (4,642,717.205 × Key passes per 90) + (3,709,531.829 × Second assists per 90) + (3,382,886.634 × Percentage of shots on target) + (3,202,644.249 × Percentage of offensive duels won) + (2,793,731.966 × Percentage of accurate passes) + (2,527,585.49 × Aerial duels per 90) + (2,468,846.23 × Assists per 90) + (2,214,208.879 × Non-penalty goals) + (2,173,518.269 × Crosses per 90) + (2,157,610.137 × xG per 90) + (1,329,497.103 × Third assists per 90) + (1,184,635.717 × Percentage of successful dribbles) + (1,156,439.669 × Progressive runs per 90) + (289,763.8726 × Matches played) + (244,354.2701 × Goals per 90) + (-96,520.5278 × Percentage of penalty conversion) + (-197,742.4933 × Received long passes per 90) + (-1,434,853.803 × Percentage of duels won) + (-13,710,073.52 × Age)
