# Supplementary material for: A data-driven performance index for center forwards in the English Premier League
Source: Sci Rep. 2026 Jun 8;16:26001. doi: 10.1038/s41598-026-55681-9 (PMC13487182; doi:10.1038/s41598-026-55681-9)
Supplement: Supplementary file 3 [file 41598_2026_55681_MOESM3_ESM.docx]

Supplementary Material IV: SPI formula — InStat Index

SPI = (3.2018 × xG per 90) + (2.0731 × Shots) + (1.8863 × Key passes per 90) + (1.8455 × Matches played) + (1.3893 × Smart passes per 90) + (1.3312 × Passes per 90) + (1.2329 × Second assists per 90) + (1.2153 × Goals) + (1.0693 × Percentage of accurate passes) + (0.9140 × Percentage of shots on target) + (0.8954 × Non-penalty goals) + (0.8762 × Aerial duels per 90) + (0.7233 × Third assists per 90) + (0.6379 × Percentage of successful dribbles) + (0.6335 × Minutes played) + (0.6004 × Percentage of duels won) + (0.5528 × Percentage of accurate forward passes) + (0.5275 × Passes to penalty area per 90) + (0.4864 × Successful attacking actions per 90) + (0.3523 × Dribbles per 90) + (0.3166 × Touches in box per 90) + (0.1995 × Progressive runs per 90) + (0.1932 × Percentage of penalty conversion) + (0.1866 × Percentage of accurate passes to final third) + (-0.0972 × Crosses per 90) + (-1.7313 × Received long passes per 90)
