# Supplementary material for: A data-driven performance index for center forwards in the English Premier League
Source: Sci Rep. 2026 Jun 8;16:26001. doi: 10.1038/s41598-026-55681-9 (PMC13487182; doi:10.1038/s41598-026-55681-9)
Supplement: Supplementary file 4 [file 41598_2026_55681_MOESM4_ESM.docx]

Supplementary Material V: SPI formula — Hybrid Index

SPI = (0.2175 × xG per 90) + (0.2028 × Shots) + (0.1815 × Key passes per 90) + (0.1208 × Matches played) + (0.11 × Goals) + (0.076 × Passes per 90) + (0.0759 × Smart passes per 90) + (0.0664 × Percentage of accurate passes) + (0.066 × Second assists per 90) + (0.0658 × Minutes played) + (0.0654 × Percentage of successful dribbles) + (0.0624 × Progressive runs per 90) + (0.0532 × Percentage of duels won) + (0.0529 × Touches in box per 90) + (0.0417 × Percentage of shots on target) + (0.0412 × Non-penalty goals) + (0.0409 × Aerial duels per 90) + (0.0368 × Third assists per 90) + (0.0356 × Percentage of accurate passes to final third) + (0.0321 × Passes to penalty area per 90) + (0.0321 × Percentage of penalty conversion) + (0.0304 × Successful attacking actions per 90) + (0.0195 × Percentage of accurate forward passes) + (0.0084 × Crosses per 90) + (0.0036 × Percentage of accurate passes to penalty area) + (-0.0636 × Received long passes per 90) + (-0.1774 × Age)
